# Vestibular paroxysmia: a treatable neurovascular cross-compression syndrome

**DOI:** 10.1007/s00415-015-7973-3

**Published:** 2016-04-15

**Authors:** Thomas Brandt, Michael Strupp, Marianne Dieterich

**Affiliations:** Institute for Clinical Neurosciences, Ludwig-Maximilians University, Munich, Germany; Department of Neurology, Ludwig-Maximilians University, Munich, Germany; German Center for Vertigo and Balance Disorders, Ludwig-Maximilians University, Munich, Germany; Munich Cluster for Systems Neurology (SyNergy), Munich, Germany

**Keywords:** Vestibular paroxysmia, Neurovascular cross-compression, Vestibular nerve, Episodic vertigo, Carbamazepine, Review

## Abstract

The leading symptoms of vestibular paroxysmia (VP) are recurrent, spontaneous, short attacks of spinning or non-spinning vertigo that generally last less than one minute and occur in a series of up to 30 or more per day. VP may manifest when arteries in the cerebellar pontine angle cause a segmental, pressure-induced dysfunction of the eighth nerve. The symptoms are usually triggered by direct pulsatile compression with ephaptic discharges, less often by conduction blocks. MR imaging reveals the neurovascular compression of the eighth nerve (3D constructive interference in steady state and 3D time-of-flight sequences) in more than 95 % of cases. A loop of the anterior inferior cerebellar artery seems to be most often involved, less so the posterior inferior cerebellar artery, the vertebral artery, or a vein. The frequent attacks of vertigo respond to carbamazepine or oxcarbazepine, even in low dosages (200–600 mg/d or 300–900 mg/d, respectively), which have been shown to also be effective in children. Alternative drugs to try are lamotrigine, phenytoin, gabapentin, topiramate or baclofen or other non-antiepileptic drugs used in trigeminal neuralgia. The results of ongoing randomized placebo-controlled treatment studies, however, are not yet available. Surgical microvascular decompression of the eighth nerve is the “ultima ratio” for medically intractable cases or in exceptional cases of non-vascular compression of the eighth nerve by a tumor or cyst. The International Barany Society for Neuro-Otology is currently working on a consensus document on the clinical criteria for establishing a diagnosis of VP as a clinical entity.

## Introduction

**Fig. 1:**
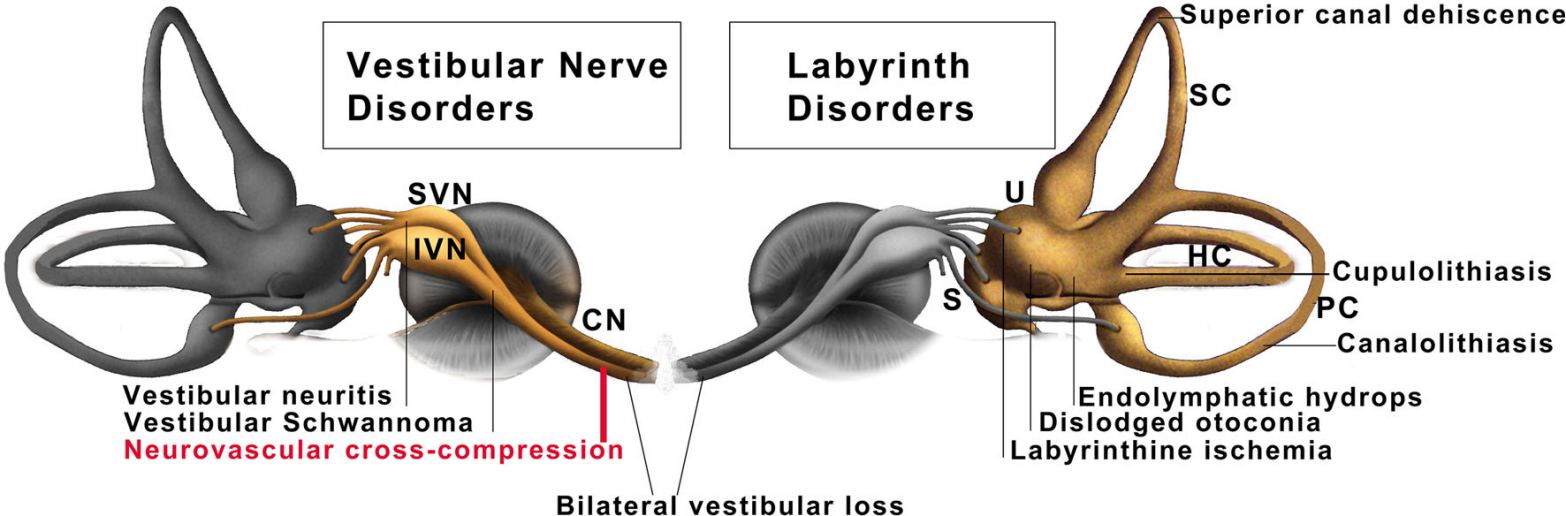
Common peripheral vertigo and balance disorders of the vestibular labyrinth (*right*) and the vestibular nerve (*left*) make up about 47 % of more than 17,000 outpatients in a multidisciplinary dizziness unit (Brandt et al. 2014). Labyrinthine disorders include benign paroxysmal positional vertigo due to canalo- or cupulolithiasis, superior canal dehiscence syndrome, and Menière’s disease with endolymphatic hydrops. Vestibular nerve disorders include superior and rare inferior vestibular neuritis, vestibular schwannoma, bilateral vestibulopathy, and vestibular paroxysmia due to neurovascular cross-compression. The frequency of vestibular paroxysmia is nearly 4 % (schematic drawing of the labyrinth modified from Leblanc). *SC, HC, PC* superior, horizontal, posterior canal, *U* utricle, *S* saccule, *SVN, IVN* superior, inferior vestibular nerve, *CN* cochlear nerve

The main symptoms of vestibular paroxysmia (VP) are brief attacks of spinning or non-spinning vertigo which lasts a fraction of a second to a few minutes and occurs with or without ear symptoms (tinnitus and hypo- or hyperacusis). Arteries or rarely veins in the cerebellar pontine angle are the pathophysiological cause of a segmental, pressure-induced dysfunction of the eighth nerve. As in trigeminal neuralgia, the symptoms are triggered by direct pulsatile compression and ephaptic pathological paroxysmal interaxonal transmission between neighbouring and possibly in part demyelinated axons [1, 2]. VP accounts for 3.7 % of 17,718 consecutive outpatients of the German Center for Vertigo and Balance Disorders (Fig. 1).

The syndrome of neurovascular cross-compression of the eighth nerve was previously connected with so-called “disabling positional vertigo” [3], a very heterogeneous syndrome of vertigo with symptoms of various durations (from seconds to days), various characteristic features (rotatory or postural vertigo, light-headedness or gait instability without vertigo), and varying accompanying symptoms. As these vague descriptions also applied to patients with benign paroxysmal positional vertigo, Menière’s disease, bilateral vestibulopathy, or somatoform phobic postural vertigo, the clinical definition was subsequently made more precise and then the term ‘vestibular paroxysmia’ was introduced [1]. Whereas Møller and Jannetta [4] recommended microvascular surgical decompression with various degrees of success, we introduced the use of anticonvulsants such as carbamazepine as the medical therapy of first choice [1].

## Clinical syndrome

VP is suspected if brief and frequent attacks of vertigo are accompanied by the following features [1]:

- Short attacks of rotatory or postural vertigo last for seconds to minutes with instability of posture and gait.
- Attacks may often be triggered by particular head positions or hyperventilation, and may be influenced by changing the head position.
- Unilateral hypoacusis or tinnitus occurs during the attack, occasionally or permanently.
- In the course of the disease, measurable vestibular and/or cochlear deficits increase during the attack but are less pronounced during the attack-free interval (neurophysiological function tests used include audiogram, acoustic-evoked potentials, caloric testing, and test for subjective visual vertical).
- Attacks are improved or lessened by administering carbamazepine (even a low dosage is effective).
- No central vestibular/ocular motor disorders or brainstem signs are present.

The International Barany Society for Neuro-Otology is currently working on a consensus document on the clinical criteria for establishing a diagnosis of VP.

Approximately 20–45 % of patients undergoing ocular motor and functional testing of the eighth cranial nerve exhibit signs of a unilateral vestibular hypofunction during headshaking nystagmus tests, the head-impulse test, and caloric irrigation [2, 5]. In some of the patients, spontaneous nystagmus can be provoked by hyperventilation [2].

Conclusions can be drawn from the type of complaints—i.e., vestibular (originating from the canals or otolith organs) or cochlear symptoms—about the portion of the nerve affected. If symptoms of various nerves are combined, the site of the lesion can possibly be deduced. Thus, for example, simultaneously occurring symptoms of the seventh and eighth cranial nerves (with contraction of the frontal muscle, vertigo and slightly staggered double images; [6]) indicate an irritation of both nerves in the internal acoustic meatus, where both lie in close proximity to each other. Finally, analogous cases with relapsing tinnitus have been described [7, 8]. It can equally be a sign of an irritation (excitation) or of a loss of function (inhibition).

There seem to be two peaks in the frequency of occurrence related to age: one peak begins at an early age in cases of vertebrobasilar vascular anomalies and a second one between the ages of 40 and 70, when vascular elongation occurs due to increasing atherosclerosis and stronger pulsations due to arterial hypertension of old age. The course is generally chronic. Men are affected twice as often as women. The condition can also manifest in children who present with the above-described symptoms sometimes together with brief bouts of nystagmus for seconds to minutes [9]. The neurovascular cross-compression syndrome may resolve spontaneously since vascular brain and bone structures grow at different speeds [10].

## Pathophysiological mechanism

**Fig. 2:**
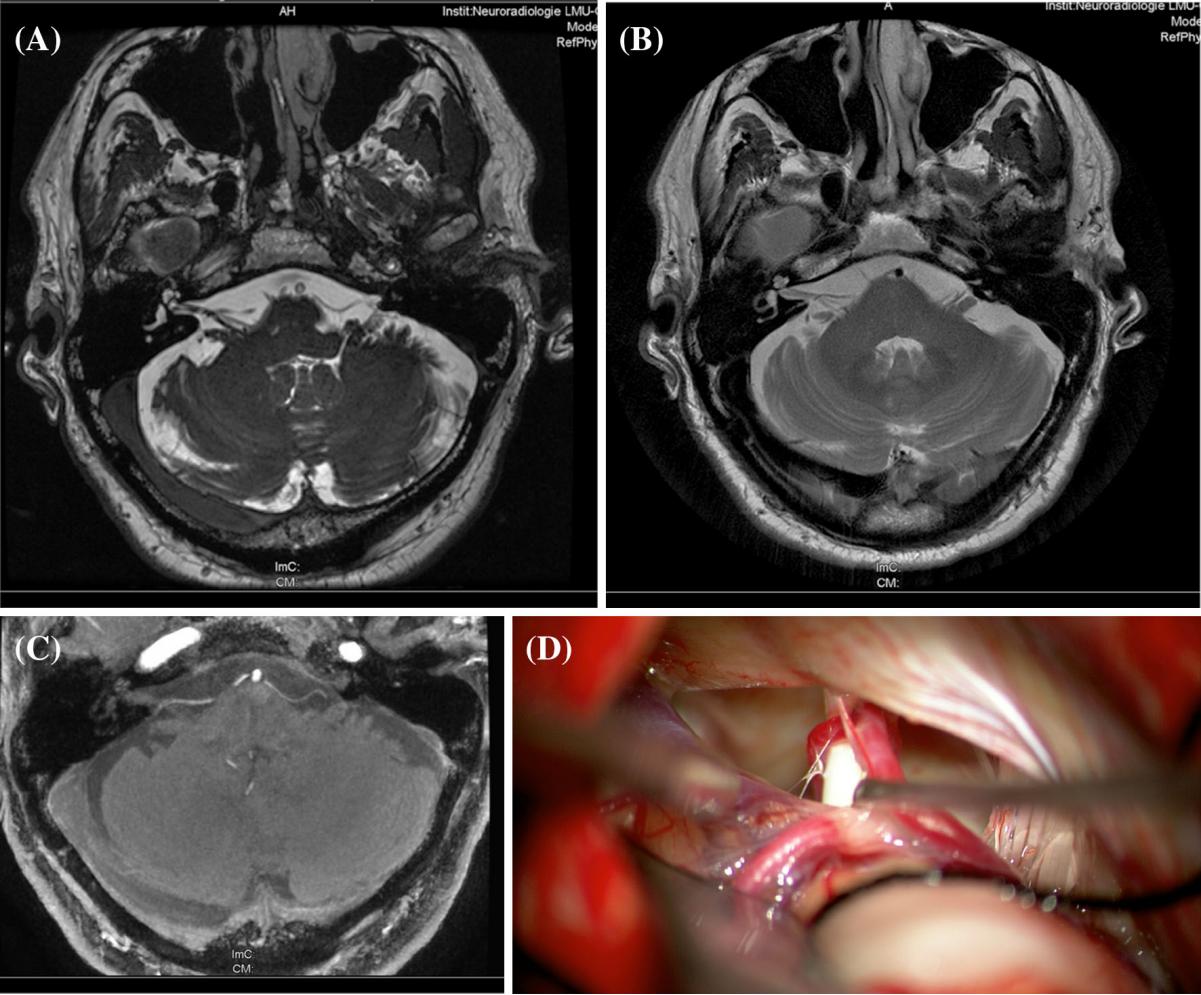
MRI of a patient with right-sided vestibular paroxysmia. **a** Fast imaging employing steady state acquisition sequence (FIESTA) and **b** T2 “propeller” and **c** time-of-flight MR-angiography demonstrate a neuro-vascular compression of the eighth cranial nerve by the AICA. This was also found intraoperatively (**d**) (modified from [23])

As in trigeminal neuralgia, hemifacial spasm, glossopharyngeal neuralgia or myokymia of the superior oblique muscle [11], it is assumed that a neurovascular cross-compression of the eighth cranial nerve is the cause of these short episodes of vertigo in VP [1, 12]. This was more recently also described in a single well-documented patient who underwent a successful operation [13] (Fig. 2). Aberrant, in part arteriosclerotically elongated and dilated, and consequently more pulsating arteries in the cerebellopontine angle are thought to be the pathophysiological cause of a segmental, pressure-induced lesion with demyelination of the central (oligodendroglia) myelin. A loop of the AICA seems to be involved most often, seldom the PICA, the vertebral artery, or a vein. The symptoms are triggered by direct pulsatile compression with ephaptic discharges or less often conduction blocks. Another cause under discussion is central hyperactivity in the vestibular nuclei, which is induced and maintained by the compression. Finally, in addition to elongation and increased looping, a vascular malformation or arterial ectasia of the posterior fossa can also cause the nerve compression. A megalodolichobasilar artery, for example, can both subsequently and simultaneously elicit multiple cranial nerve paroxysmias like VP, trigeminal neuralgia, and hemifacial spasm [14].

**Fig. 3:**
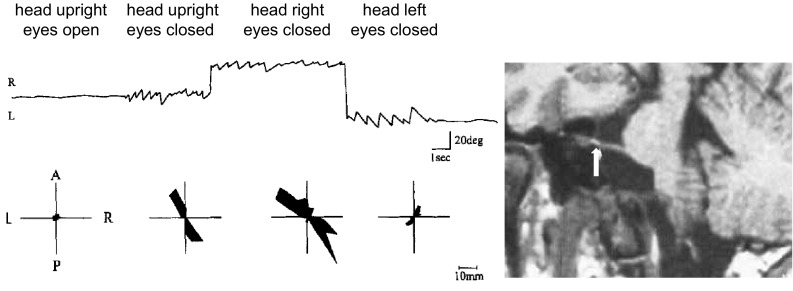
Episodes of vestibular nerve paroxysmia alternating with conduction block failure. Recordings of eye movements (*top*) and postural sway (*bottom*) during head rotation in a patient with eighth nerve compression by a cerebellopontine angle cyst. The stretched nerve is shown in the MRI (*see arrow*). Electronystagmography showed eye movements with head straight (eyes open, eyes closed) and head rotation to the *right* or *left*. Note the directional change of the left-beating nystagmus to the right with head turned left. The simultaneous body sway measured by posturography is shown in the *bottom line*. Eye closure and head turn to the right increased diagonal body sway from left-forward to right-backward, while head rotation to the left led to a decrease of body sway with a 90° shift of the preferred direction (modified from Arbusow et al. 1998)

Occasionally vertigo attacks lasting seconds and caused by head movements point to an arachnoid cyst that stretches the vestibulocochlear nerve [15]. This pathogenesis can result in a combination of longer-lasting vestibular hypofunction due to conduction-block symptoms in one direction (hours to days) and paroxysmal vestibular excitations (for seconds) in the opposite direction elicited by head movements (Fig. 3). This represents a head-position dependent transition from conduction block to ectopic discharges, which have also been observed when peripheral nerves are compressed [15]. The same mechanisms hold for VP due to neurovascular cross-compression. A possible change in the direction of vertigo, nystagmus, and body sway during an attack of VP makes it difficult to determine which side is affected. This is especially true when surgery has to be considered in medically unresponsive cases or when evaluating different vestibular functions such as caloric irrigation, ocular torsion, tilt of subjective visual vertical, and vestibular evoked myogenic potentials [5]. Auditory symptoms (tinnitus or hearing loss) during the attack provide a more reliable indication of the affected side.

## Imaging neurovascular cross-compression of the eighth nerve

Signs of an arterial (rarely venous) compression of the eighth cranial nerve are visible in MRI (3D constructive interference in steady state, CISS, and 3D time-of-flight sequences, TOF; Fig. 2). In a study on 32 patients with VP neurovascular compression in the terminal area of the vestibulocochlear nerve was detected in 95 % of the affected patients. In this series a bilateral neurovascular compression was found in 42 % of the patients [2]. Such neurovascular compressions are known to also occur in healthy subjects, but they can be diagnosed as pathological only if the corresponding accompanying clinical symptoms are present. Nevertheless, larger prospective clinical studies are still lacking as to how often such neurovascular contacts can also be imaged in healthy subjects. In a control group of patients with trigeminal neuralgia an asymptomatic contact of the eighth nerve was found in about 35 % [5].

The distance of the affected, most vulnerable region of the myelin sheath of the vestibulocochlear nerve from the nerve exit zone out of the brainstem has been measured in millimeters (between 0.0 and 10.2 mm; [5]). Therefore, it can be assumed that it is the long intracisternal course, which is surrounded by central myelin of the oligodendrocytes, for it corresponds to the first 10–15 mm after the nerve exits [16].

High-resolution diffusion tensor imaging of patients with trigeminal neuralgia revealed a significantly lower anisotropy and a higher apparent diffusion coefficient in the affected trigeminal root, which correlates with structural atrophic nerve changes [17]. Comparable findings are not yet available for the eighth nerve because of methodological limitations due to the short course of the eighth nerve from the brainstem to the internal acoustic meatus and the adjacent temporal bone. A 7-Tesla MRI in six patients with VP did not exhibit any structural abnormalities. This finding supports the view that high-field MRI does not help differentiate between symptomatic and asymptomatic neurovascular cross-compression in VP [18].

Nevertheless, a cranial MRI should be performed to exclude the presence of a tumor in the area of the cerebellar pontine angle, arachnoid cysts, megalodolichobasilar artery, lacunar infarctions or brainstem plaques due to MS (paroxysmal brainstem attacks) or other lesions.

## Treatment

So far there are no data available from randomized prospective placebo-controlled studies on the efficacy of various drugs in VP. Theoretically, one would expect blockers of the fast sodium channel such as carbamazepine or oxcarbazepine to be effective as they are in other diseases caused by neurovascular cross-compression. Observational studies have shown that both agents were effective in low dosages of 200–600 mg/day of carbamazepine and 300–900 mg/day of oxcarbazepine. Furthermore, a randomized controlled trial currently being analyzed showed that oxcarbazepine reduces the days with vertigo compared to placebo, but the drop-out rate was >50 % [19]. Until the results of the ongoing randomized placebo-controlled trial (VESPA) are available, we recommend the following pragmatic therapy [20].

## Medical treatment

A therapeutic approach with a low dosage of

- Carbamazepine (200–600 mg/day) or
- Oxcarbazepine (300–900 mg/day)

is expedient, and moreover a positive response is diagnostic. A study on the course of the disease in 32 patients over a 3-year period revealed a significant and continuing decrease in the attack frequency down to 10 % of the initial value as well as a reduction in the intensity and duration of the attacks [2].

There is very limited evidence for the use of lamotrigine, baclofen, topiramate [21, 22] or non-antiepileptic drugs which have been tried in trigeminal neuralgia [23] or for gabapentin in VP [7].

## Surgical treatment

Despite the report of partial successes [12] and a clinically well-documented single case [13], operative microvascular decompression should only be considered in medically intractable cases. On the one hand, there is the risk of a brainstem infarction due to intra- or post-operative vasospasm (up to 3–5 %), and, on the other as stated above, it is difficult to determine the affected side with enough certainty. However, if there are additional causes, such as the above-mentioned arachnoid cyst in the cerebellar pontine angle, the operation is recommended, for drug therapy only rarely leads to the absence of symptoms.

## Differential diagnosis and clinical problems

Important differential diagnoses are:

- Paroxysmal brainstem attacks,
- Superior oblique myokymia,
- Vestibular migraine,
- Benign paroxysmal positional vertigo (BPPV),
- Somatoform phobic postural vertigo (functional dizziness),
- Rotational vertebral artery occlusion syndrome,
- Superior canal dehiscence syndrome,
- Central positional/positioning nystagmus,
- Panic attacks,
- Orthostatic dysregulation,
- Epileptic vestibular aura.

The differential diagnosis is generally straightforward, because of the characteristic brevity (seconds up to a few minutes, very seldom many hours) and the frequently recurring attacks of vertigo. Only paroxysmal brainstem attacks with vertigo, dysarthria, and ataxia can be difficult to distinguish, as they too respond to low dosages of carbamazepine. It has been shown that they are caused by a midbrain lesion due to MS plaques or lacunar infarctions [24], which also lead to ephaptic discharges of neighbouring fibres of the brainstem paths. In such cases the use of MRI with thin brainstem slices is expedient for establishing the diagnosis. Another cranial nerve compression syndrome, superior oblique myokymia, can be easily differentiated, since it causes frequent attacks of monocular vertical-rotatory oscillopsia. The physician and patient can observe this by covering one eye. Spontaneous episodic vertigo in vestibular migraine rarely lasts for seconds and does not occur in a series of multiple attacks per day. BPPV due to canalolithiasis can be diagnosed by the typical crescendo–decrescendo nystagmus caused by the positioning manoeuvre. These typical features are not seen in VP and are not triggered as regularly by positioning. Head movement-induced apparent perturbations of the body, very like those in VP, may occur in phobic postural vertigo. However, these sensations occur on top of a chronic subjective unsteadiness with a fear of falling without falls. The same is true for other forms of functional dizziness [25] which have a high psychiatric comorbidity, especially anxiety disorders and depression [26]. The vertebral artery occlusion syndrome may start with a short vertigo attack with nystagmus and ataxia regularly elicited by head rotations in one direction which occlude the dominant artery of the opposite side. The symptoms of the superior canal dehiscence syndrome may mimic VP but are elicited by intracranial pressure changes (Valsalva manoeuvre) or acoustic stimulation (Tullio phenomenon). Rare central positional nystagmus syndromes mostly require changes in the position of the head relative to gravity. They last as long as the position is maintained and often manifest without any vertigo or with only weak vertigo. Panic attacks often last longer than typical attacks of VP. It may be helpful to ask the patient which of the symptoms (vertigo or anxiety) appears first in order to differentiate between the two. In orthostatic hypotension the symptoms occur when the patients stand up and may be associated with vertigo and nystagmus; the key to this diagnosis is an examination with the Schellong or squatting-standing tests. Epileptic vestibular auras can manifest with short attacks of vertigo and nystagmus, but most of these patients are known to have epilepsy.

Desirable clinical developments for the management of VP in the future include:

- improved imaging techniques to reveal neurovascular cross-compression of the eighth nerve, especially to distinguish between symptomatic and asymptomatic contacts,
- clinical tests to reliably determine the affected side,
- randomized placebo-controlled trials,
- new medications.
